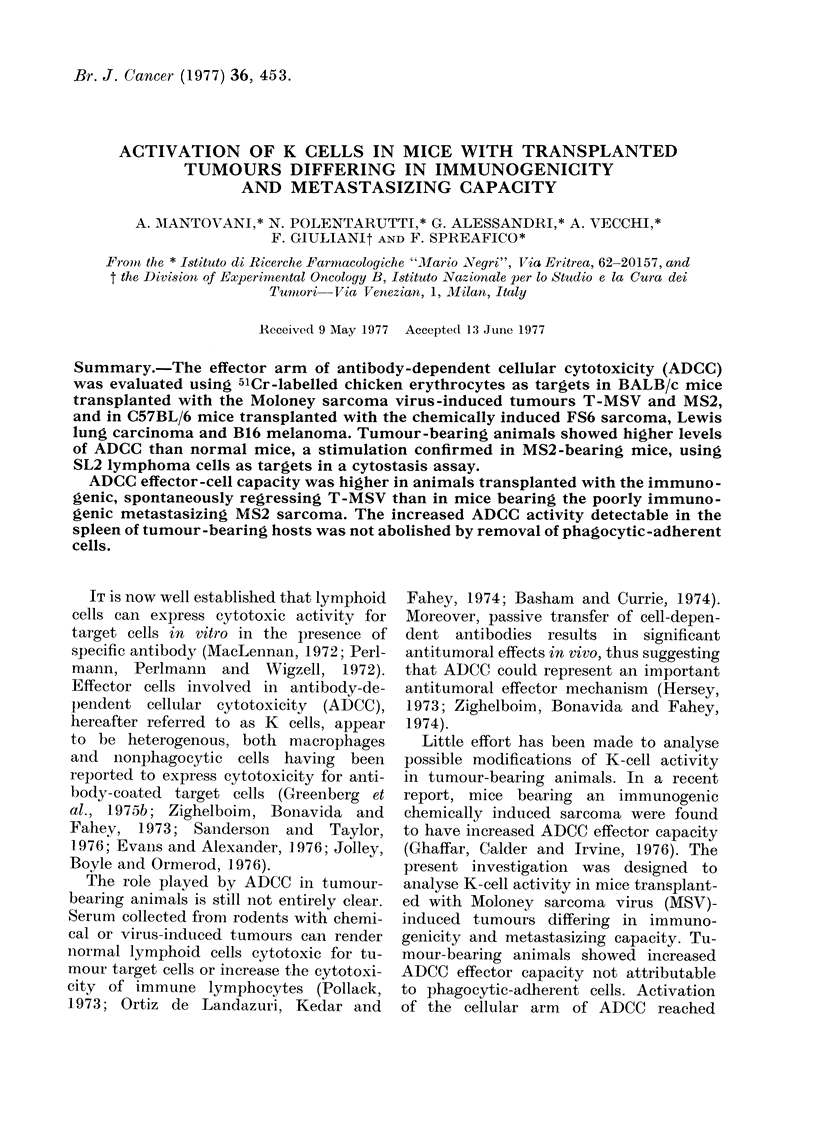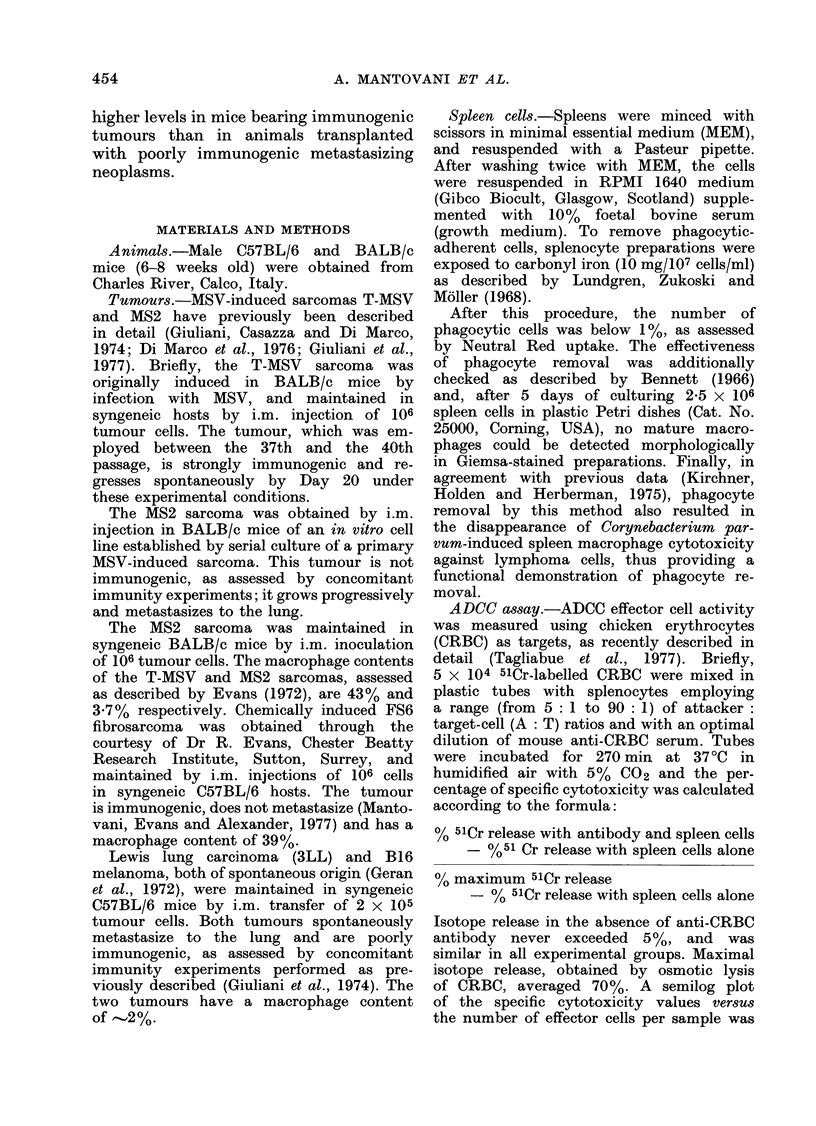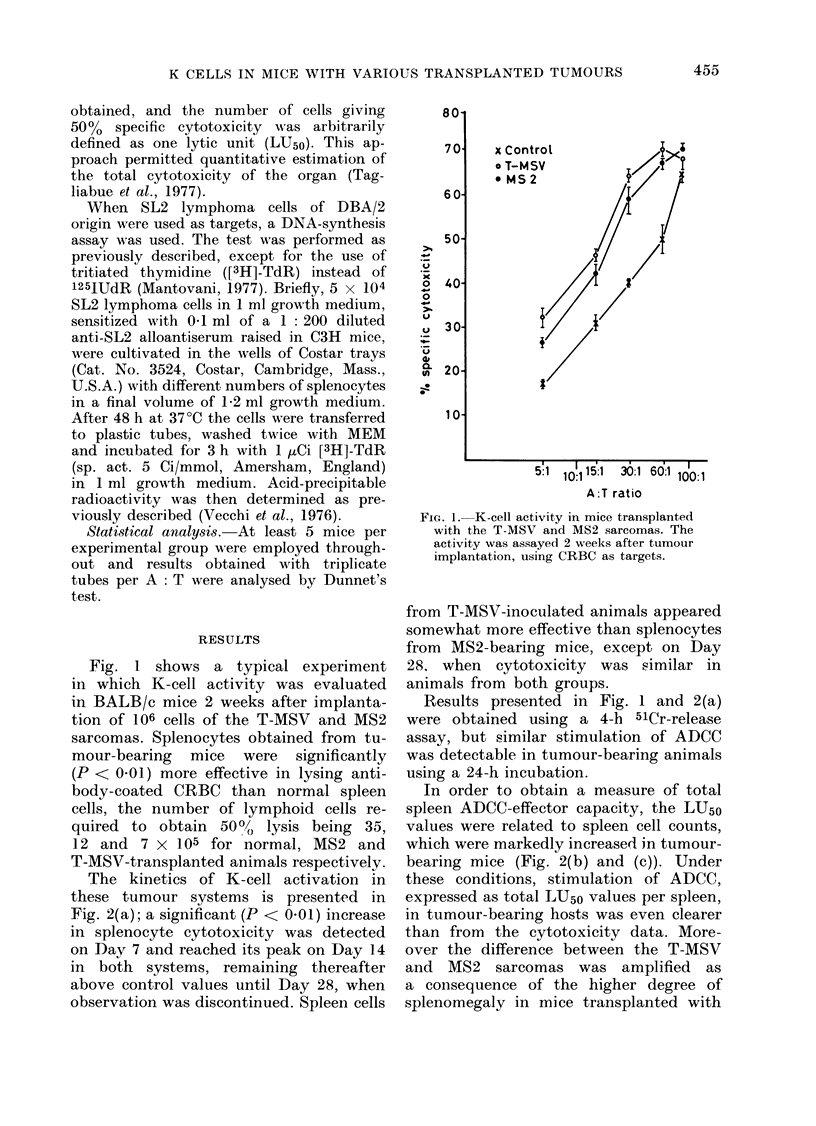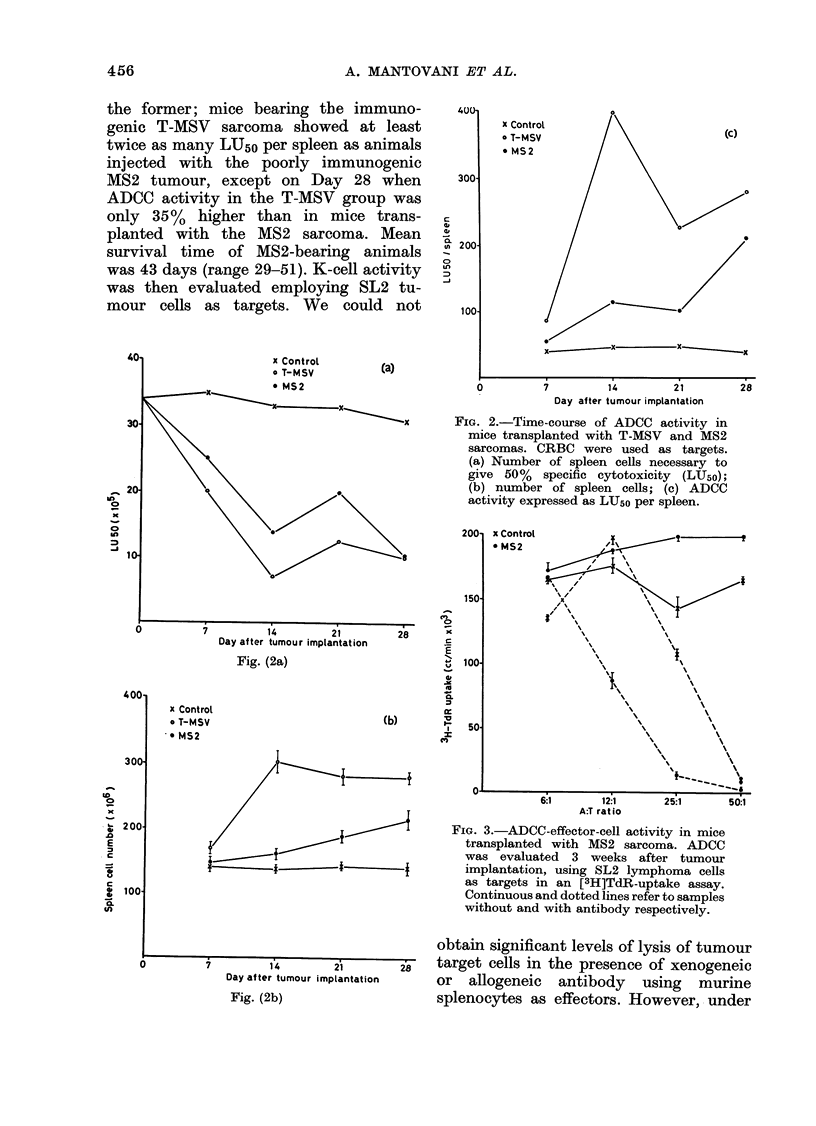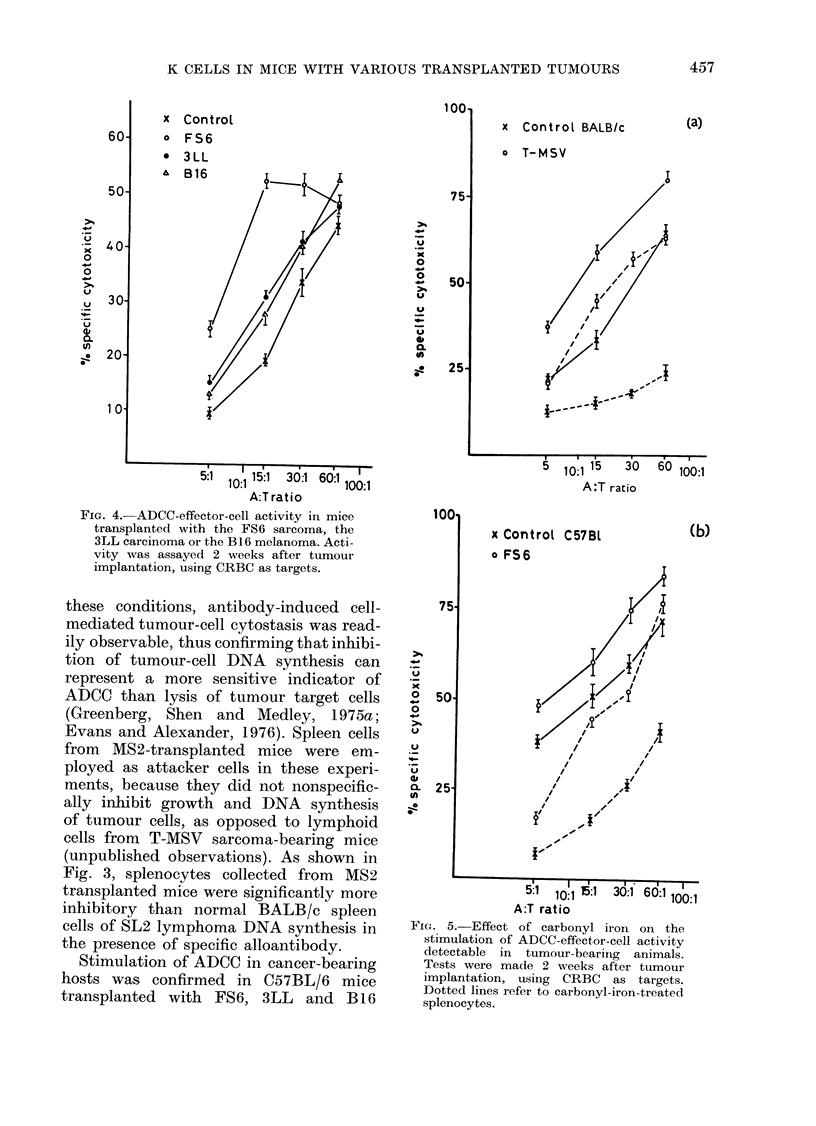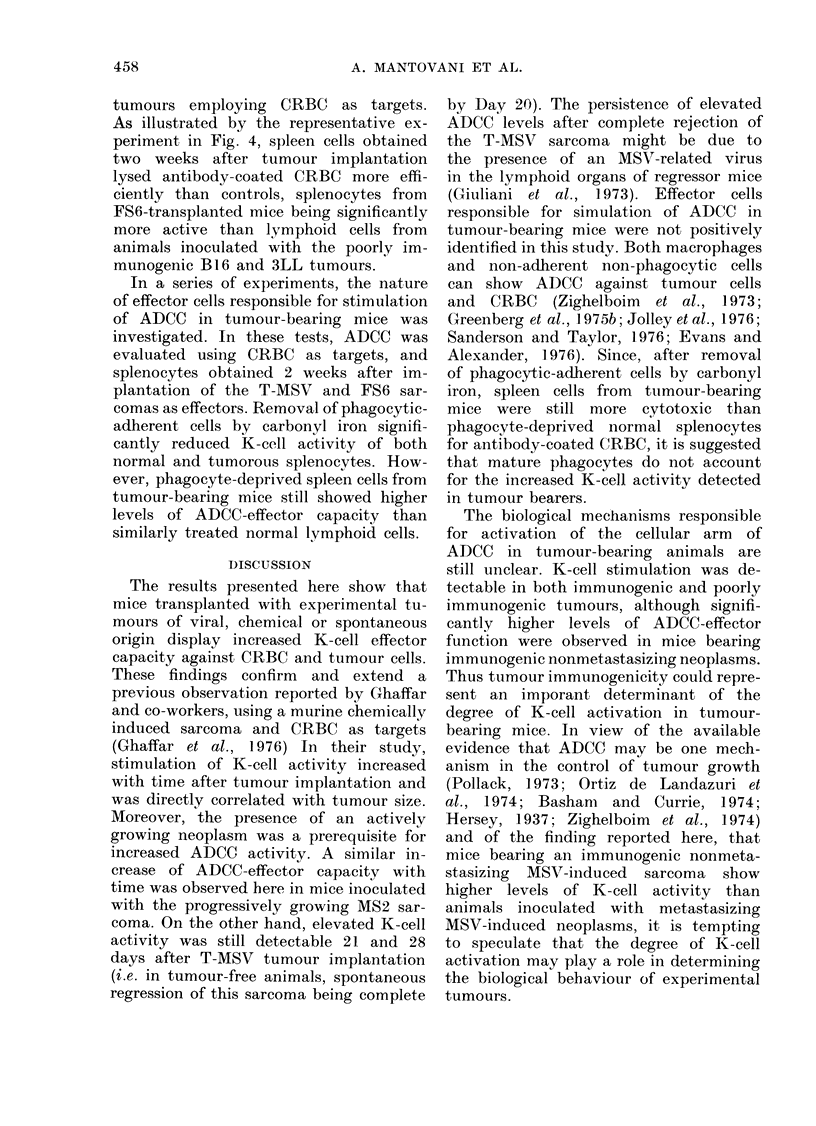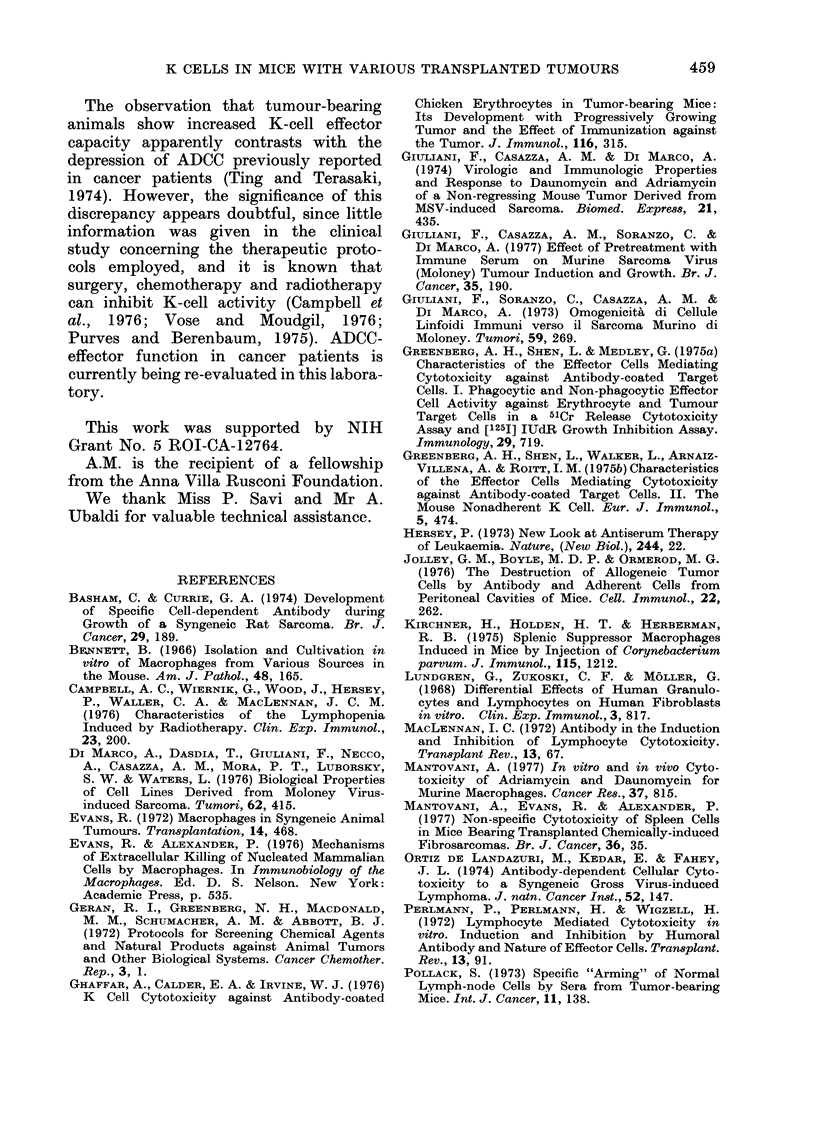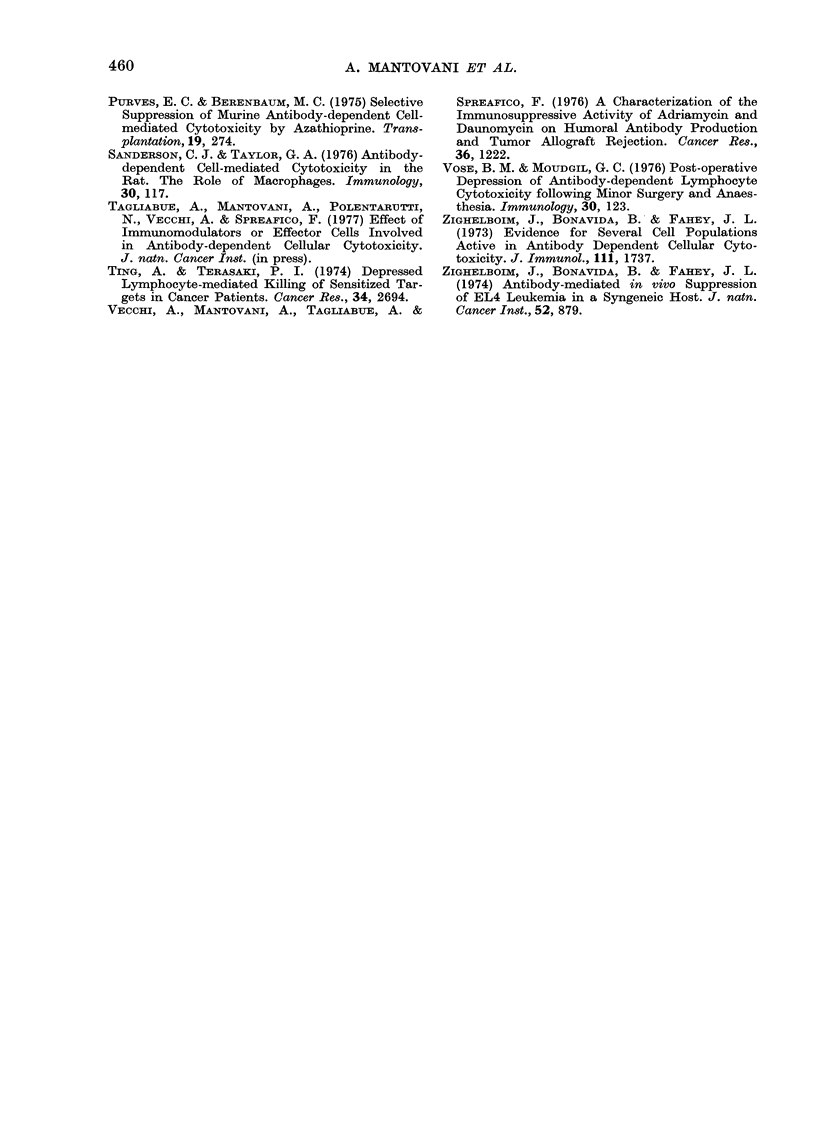# Activation of K cells in mice with transplanted tumours differing in immunogenicity and metastasizing capacity.

**DOI:** 10.1038/bjc.1977.214

**Published:** 1977-10

**Authors:** A. Mantovani, N. Polentarutti, G. Alessandri, A. Vecchi, F. Giuliani, F. Spreafico

## Abstract

The effector arm of antibody-dependent cellular cytotoxicity (ADCC) was evaluated using 51Cr-labelled chicken erythrocytes as targets in BALB/c mice transplanted with the Moloney sarcoma virus-induced tumours T-MSV and MS2, and in C57BL/6 mice transplanted with the chemically induced FS6 sarcoma, Lewis lung carcinoma and B16 melanoma. Tumour-bearing animals showed higher levels of ADCC than normal mice, a stimulation confirmed in MS2-bearing mice, using SL2 lymphoma cells as targets in a cytostasis assay. ADCC effector-cell capacity was higher in animals transplanted with the immunogenic, spontaneously regressing T-MSV than in mice bearing the poorly immunogenic metastasizing MS2 sarcoma. The increased ADCC activity detectable in the spleen of tumour-bearing hosts was not abolished by removal of phagocytic-adherent cells.


*Br. J. Cancer* (1977) **36**, 453.

### ACTIVATION OF K CELLS IN MICE WITH TRANSPLANTED TUMOURS DIFFERING IN IMMUNOGENICITY AND METASTASIZING CAPACITY

A. MANTOVANI,* N. POLENTARUTTI,* G. ALESSANDRI,* A. VECCHI,* F. GIULIANI† AND F. SPREAFICO*

*From the * Istituto di Ricerche Farmacologiche "Mario Negri", Via Eritrea, 62–20157, and † the Division of Experimental Oncology B, Istituto Nazionale per lo Studio e la Cura dei Tumori—Via Venezian, 1, Milan, Italy*

Received 9 May 1977 Accepted 13 June 1977

**Summary.—The effector arm of antibody-dependent cellular cytotoxicity (ADCC) was evaluated using $^{51}$Cr-labelled chicken erythrocytes as targets in BALB/c mice transplanted with the Moloney sarcoma virus-induced tumours T-MSV and MS2, and in C57BL/6 mice transplanted with the chemically induced FS6 sarcoma, Lewis lung carcinoma and B16 melanoma. Tumour-bearing animals showed higher levels of ADCC than normal mice, a stimulation confirmed in MS2-bearing mice, using SL2 lymphoma cells as targets in a cytostasis assay.**

**ADCC effector-cell capacity was higher in animals transplanted with the immunogenic, spontaneously regressing T-MSV than in mice bearing the poorly immunogenic metastasizing MS2 sarcoma. The increased ADCC activity detectable in the spleen of tumour-bearing hosts was not abolished by removal of phagocytic-adherent cells.**

IT is now well established that lymphoid cells can express cytotoxic activity for target cells *in vitro* in the presence of specific antibody (MacLennan, 1972; Perlmann, Perlmann and Wigzell, 1972). Effector cells involved in antibody-dependent cellular cytotoxicity (ADCC), hereafter referred to as K cells, appear to be heterogenous, both macrophages and nonphagocytic cells having been reported to express cytotoxicity for antibody-coated target cells (Greenberg *et al.*, 1975*b*; Zighelboim, Bonavida and Fahey, 1973; Sanderson and Taylor, 1976; Evans and Alexander, 1976; Jolley, Boyle and Ormerod, 1976).

The role played by ADCC in tumour-bearing animals is still not entirely clear. Serum collected from rodents with chemical or virus-induced tumours can render normal lymphoid cells cytotoxic for tumour target cells or increase the cytotoxicity of immune lymphocytes (Pollack, 1973; Ortiz de Landazuri, Kedar and Fahey, 1974; Basham and Currie, 1974). Moreover, passive transfer of cell-dependent antibodies results in significant antitumoral effects *in vivo*, thus suggesting that ADCC could represent an important antitumoral effector mechanism (Hersey, 1973; Zighelboim, Bonavida and Fahey, 1974).

Little effort has been made to analyse possible modifications of K-cell activity in tumour-bearing animals. In a recent report, mice bearing an immunogenic chemically induced sarcoma were found to have increased ADCC effector capacity (Ghaffar, Calder and Irvine, 1976). The present investigation was designed to analyse K-cell activity in mice transplanted with Moloney sarcoma virus (MSV)-induced tumours differing in immunogenicity and metastasizing capacity. Tumour-bearing animals showed increased ADCC effector capacity not attributable to phagocytic-adherent cells. Activation of the cellular arm of ADCC reached higher levels in mice bearing immunogenic tumours than in animals transplanted with poorly immunogenic metastasizing neoplasms.

### MATERIALS AND METHODS

*Animals.*—Male C57BL/6 and BALB/c mice (6–8 weeks old) were obtained from Charles River, Calco, Italy.

*Tumours.*—MSV-induced sarcomas T-MSV and MS2 have previously been described in detail (Giuliani, Casazza and Di Marco, 1974; Di Marco *et al.*, 1976; Giuliani *et al.*, 1977). Briefly, the T-MSV sarcoma was originally induced in BALB/c mice by infection with MSV, and maintained in syngeneic hosts by i.m. injection of $10^6$ tumour cells. The tumour, which was employed between the 37th and the 40th passage, is strongly immunogenic and regresses spontaneously by Day 20 under these experimental conditions.

The MS2 sarcoma was obtained by i.m. injection in BALB/c mice of an *in vitro* cell line established by serial culture of a primary MSV-induced sarcoma. This tumour is not immunogenic, as assessed by concomitant immunity experiments; it grows progressively and metastasizes to the lung.

The MS2 sarcoma was maintained in syngeneic BALB/c mice by i.m. inoculation of $10^6$ tumour cells. The macrophage contents of the T-MSV and MS2 sarcomas, assessed as described by Evans (1972), are 43% and 3·7% respectively. Chemically induced FS6 fibrosarcoma was obtained through the courtesy of Dr R. Evans, Chester Beatty Research Institute, Sutton, Surrey, and maintained by i.m. injections of $10^6$ cells in syngeneic C57BL/6 hosts. The tumour is immunogenic, does not metastasize (Mantovani, Evans and Alexander, 1977) and has a macrophage content of 39%.

Lewis lung carcinoma (3LL) and B16 melanoma, both of spontaneous origin (Geran *et al.*, 1972), were maintained in syngeneic C57BL/6 mice by i.m. transfer of $2 \times 10^5$ tumour cells. Both tumours spontaneously metastasize to the lung and are poorly immunogenic, as assessed by concomitant immunity experiments performed as previously described (Giuliani *et al.*, 1974). The two tumours have a macrophage content of ~2%.

*Spleen cells.*—Spleens were minced with scissors in minimal essential medium (MEM), and resuspended with a Pasteur pipette. After washing twice with MEM, the cells were resuspended in RPMI 1640 medium (Gibco Biocult, Glasgow, Scotland) supplemented with 10% foetal bovine serum (growth medium). To remove phagocytic-adherent cells, splenocyte preparations were exposed to carbonyl iron (10 mg/$10^7$ cells/ml) as described by Lundgren, Zukoski and Möller (1968).

After this procedure, the number of phagocytic cells was below 1%, as assessed by Neutral Red uptake. The effectiveness of phagocyte removal was additionally checked as described by Bennett (1966) and, after 5 days of culturing $2{\cdot}5 \times 10^6$ spleen cells in plastic Petri dishes (Cat. No. 25000, Corning, USA), no mature macrophages could be detected morphologically in Giemsa-stained preparations. Finally, in agreement with previous data (Kirchner, Holden and Herberman, 1975), phagocyte removal by this method also resulted in the disappearance of *Corynebacterium parvum*-induced spleen macrophage cytotoxicity against lymphoma cells, thus providing a functional demonstration of phagocyte removal.

*ADCC assay.*—ADCC effector cell activity was measured using chicken erythrocytes (CRBC) as targets, as recently described in detail (Tagliabue *et al.*, 1977). Briefly, $5 \times 10^4$ $^{51}$Cr-labelled CRBC were mixed in plastic tubes with splenocytes employing a range (from 5 : 1 to 90 : 1) of attacker : target-cell (A : T) ratios and with an optimal dilution of mouse anti-CRBC serum. Tubes were incubated for 270 min at 37°C in humidified air with 5% $CO_2$ and the percentage of specific cytotoxicity was calculated according to the formula:

$$\frac{\%\ ^{51}\text{Cr release with antibody and spleen cells} - \%\ ^{51}\text{Cr release with spleen cells alone}}{\%\ \text{maximum}\ ^{51}\text{Cr release} - \%\ ^{51}\text{Cr release with spleen cells alone}}$$

Isotope release in the absence of anti-CRBC antibody never exceeded 5%, and was similar in all experimental groups. Maximal isotope release, obtained by osmotic lysis of CRBC, averaged 70%. A semilog plot of the specific cytotoxicity values *versus* the number of effector cells per sample was obtained, and the number of cells giving 50% specific cytotoxicity was arbitrarily defined as one lytic unit ($LU_{50}$). This approach permitted quantitative estimation of the total cytotoxicity of the organ (Tagliabue *et al.*, 1977).

When SL2 lymphoma cells of DBA/2 origin were used as targets, a DNA-synthesis assay was used. The test was performed as previously described, except for the use of tritiated thymidine ([$^3$H]-TdR) instead of $^{125}$IUdR (Mantovani, 1977). Briefly, $5 \times 10^4$ SL2 lymphoma cells in 1 ml growth medium, sensitized with 0·1 ml of a 1 : 200 diluted anti-SL2 alloantiserum raised in C3H mice, were cultivated in the wells of Costar trays (Cat. No. 3524, Costar, Cambridge, Mass., U.S.A.) with different numbers of splenocytes in a final volume of 1·2 ml growth medium. After 48 h at 37 °C the cells were transferred to plastic tubes, washed twice with MEM and incubated for 3 h with 1 $\mu$Ci [$^3$H]-TdR (sp. act. 5 Ci/mmol, Amersham, England) in 1 ml growth medium. Acid-precipitable radioactivity was then determined as previously described (Vecchi *et al.*, 1976).

*Statistical analysis.*—At least 5 mice per experimental group were employed throughout and results obtained with triplicate tubes per A : T were analysed by Dunnet's test.

Fig. 1.—K-cell activity in mice transplanted with the T-MSV and MS2 sarcomas. The activity was assayed 2 weeks after tumour implantation, using CRBC as targets.

### RESULTS

Fig. 1 shows a typical experiment in which K-cell activity was evaluated in BALB/c mice 2 weeks after implantation of $10^6$ cells of the T-MSV and MS2 sarcomas. Splenocytes obtained from tumour-bearing mice were significantly ($P < 0{\cdot}01$) more effective in lysing antibody-coated CRBC than normal spleen cells, the number of lymphoid cells required to obtain 50% lysis being 35, 12 and $7 \times 10^5$ for normal, MS2 and T-MSV-transplanted animals respectively.

The kinetics of K-cell activation in these tumour systems is presented in Fig. 2(a); a significant ($P < 0{\cdot}01$) increase in splenocyte cytotoxicity was detected on Day 7 and reached its peak on Day 14 in both systems, remaining thereafter above control values until Day 28, when observation was discontinued. Spleen cells from T-MSV-inoculated animals appeared somewhat more effective than splenocytes from MS2-bearing mice, except on Day 28, when cytotoxicity was similar in animals from both groups.

Results presented in Fig. 1 and 2(a) were obtained using a 4-h $^{51}$Cr-release assay, but similar stimulation of ADCC was detectable in tumour-bearing animals using a 24-h incubation.

In order to obtain a measure of total spleen ADCC-effector capacity, the $LU_{50}$ values were related to spleen cell counts, which were markedly increased in tumour-bearing mice (Fig. 2(b) and (c)). Under these conditions, stimulation of ADCC, expressed as total $LU_{50}$ values per spleen, in tumour-bearing hosts was even clearer than from the cytotoxicity data. Moreover the difference between the T-MSV and MS2 sarcomas was amplified as a consequence of the higher degree of splenomegaly in mice transplanted with the former; mice bearing the immunogenic T-MSV sarcoma showed at least twice as many $LU_{50}$ per spleen as animals injected with the poorly immunogenic MS2 tumour, except on Day 28 when ADCC activity in the T-MSV group was only 35% higher than in mice transplanted with the MS2 sarcoma. Mean survival time of MS2-bearing animals was 43 days (range 29–51). K-cell activity was then evaluated employing SL2 tumour cells as targets. We could not obtain significant levels of lysis of tumour target cells in the presence of xenogeneic or allogeneic antibody using murine splenocytes as effectors. However, under

FIG. 2.—Time-course of ADCC activity in mice transplanted with T-MSV and MS2 sarcomas. CRBC were used as targets. (a) Number of spleen cells necessary to give 50% specific cytotoxicity ($LU_{50}$); (b) number of spleen cells; (c) ADCC activity expressed as $LU_{50}$ per spleen.

Fig. (2a)

Fig. (2b)

FIG. 3.—ADCC-effector-cell activity in mice transplanted with MS2 sarcoma. ADCC was evaluated 3 weeks after tumour implantation, using SL2 lymphoma cells as targets in an [$^3$H]TdR-uptake assay. Continuous and dotted lines refer to samples without and with antibody respectively.

FIG. 4.—ADCC-effector-cell activity in mice transplanted with the FS6 sarcoma, the 3LL carcinoma or the B16 melanoma. Activity was assayed 2 weeks after tumour implantation, using CRBC as targets.

these conditions, antibody-induced cell-mediated tumour-cell cytostasis was readily observable, thus confirming that inhibition of tumour-cell DNA synthesis can represent a more sensitive indicator of ADCC than lysis of tumour target cells (Greenberg, Shen and Medley, 1975*a*; Evans and Alexander, 1976). Spleen cells from MS2-transplanted mice were employed as attacker cells in these experiments, because they did not nonspecifically inhibit growth and DNA synthesis of tumour cells, as opposed to lymphoid cells from T-MSV sarcoma-bearing mice (unpublished observations). As shown in Fig. 3, splenocytes collected from MS2 transplanted mice were significantly more inhibitory than normal BALB/c spleen cells of SL2 lymphoma DNA synthesis in the presence of specific alloantibody.

Stimulation of ADCC in cancer-bearing hosts was confirmed in C57BL/6 mice transplanted with FS6, 3LL and B16

FIG. 5.—Effect of carbonyl iron on the stimulation of ADCC-effector-cell activity detectable in tumour-bearing animals. Tests were made 2 weeks after tumour implantation, using CRBC as targets. Dotted lines refer to carbonyl-iron-treated splenocytes.

tumours employing CRBC as targets. As illustrated by the representative experiment in Fig. 4, spleen cells obtained two weeks after tumour implantation lysed antibody-coated CRBC more efficiently than controls, splenocytes from FS6-transplanted mice being significantly more active than lymphoid cells from animals inoculated with the poorly immunogenic B16 and 3LL tumours.

In a series of experiments, the nature of effector cells responsible for stimulation of ADCC in tumour-bearing mice was investigated. In these tests, ADCC was evaluated using CRBC as targets, and splenocytes obtained 2 weeks after implantation of the T-MSV and FS6 sarcomas as effectors. Removal of phagocytic-adherent cells by carbonyl iron significantly reduced K-cell activity of both normal and tumorous splenocytes. However, phagocyte-deprived spleen cells from tumour-bearing mice still showed higher levels of ADCC-effector capacity than similarly treated normal lymphoid cells.

### DISCUSSION

The results presented here show that mice transplanted with experimental tumours of viral, chemical or spontaneous origin display increased K-cell effector capacity against CRBC and tumour cells. These findings confirm and extend a previous observation reported by Ghaffar and co-workers, using a murine chemically induced sarcoma and CRBC as targets (Ghaffar *et al.*, 1976) In their study, stimulation of K-cell activity increased with time after tumour implantation and was directly correlated with tumour size. Moreover, the presence of an actively growing neoplasm was a prerequisite for increased ADCC activity. A similar increase of ADCC-effector capacity with time was observed here in mice inoculated with the progressively growing MS2 sarcoma. On the other hand, elevated K-cell activity was still detectable 21 and 28 days after T-MSV tumour implantation (*i.e.* in tumour-free animals, spontaneous regression of this sarcoma being complete by Day 20). The persistence of elevated ADCC levels after complete rejection of the T-MSV sarcoma might be due to the presence of an MSV-related virus in the lymphoid organs of regressor mice (Giuliani *et al.*, 1973). Effector cells responsible for simulation of ADCC in tumour-bearing mice were not positively identified in this study. Both macrophages and non-adherent non-phagocytic cells can show ADCC against tumour cells and CRBC (Zighelboim *et al.*, 1973; Greenberg *et al.*, 1975*b*; Jolley *et al.*, 1976; Sanderson and Taylor, 1976; Evans and Alexander, 1976). Since, after removal of phagocytic-adherent cells by carbonyl iron, spleen cells from tumour-bearing mice were still more cytotoxic than phagocyte-deprived normal splenocytes for antibody-coated CRBC, it is suggested that mature phagocytes do not account for the increased K-cell activity detected in tumour bearers.

The biological mechanisms responsible for activation of the cellular arm of ADCC in tumour-bearing animals are still unclear. K-cell stimulation was detectable in both immunogenic and poorly immunogenic tumours, although significantly higher levels of ADCC-effector function were observed in mice bearing immunogenic nonmetastasizing neoplasms. Thus tumour immunogenicity could represent an imporant determinant of the degree of K-cell activation in tumour-bearing mice. In view of the available evidence that ADCC may be one mechanism in the control of tumour growth (Pollack, 1973; Ortiz de Landazuri *et al.*, 1974; Basham and Currie, 1974; Hersey, 1937; Zighelboim *et al.*, 1974) and of the finding reported here, that mice bearing an immunogenic nonmetastasizing MSV-induced sarcoma show higher levels of K-cell activity than animals inoculated with metastasizing MSV-induced neoplasms, it is tempting to speculate that the degree of K-cell activation may play a role in determining the biological behaviour of experimental tumours.

The observation that tumour-bearing animals show increased K-cell effector capacity apparently contrasts with the depression of ADCC previously reported in cancer patients (Ting and Terasaki, 1974). However, the significance of this discrepancy appears doubtful, since little information was given in the clinical study concerning the therapeutic protocols employed, and it is known that surgery, chemotherapy and radiotherapy can inhibit K-cell activity (Campbell *et al.*, 1976; Vose and Moudgil, 1976; Purves and Berenbaum, 1975). ADCC-effector function in cancer patients is currently being re-evaluated in this laboratory.

This work was supported by NIH Grant No. 5 ROI-CA-12764.

A.M. is the recipient of a fellowship from the Anna Villa Rusconi Foundation.

We thank Miss P. Savi and Mr A. Ubaldi for valuable technical assistance.

### REFERENCES

BASHAM, C. & CURRIE, G. A. (1974) Development of Specific Cell-dependent Antibody during Growth of a Syngeneic Rat Sarcoma. *Br. J. Cancer*, **29**, 189.

BENNETT, B. (1966) Isolation and Cultivation *in vitro* of Macrophages from Various Sources in the Mouse. *Am. J. Pathol.*, **48**, 165.

CAMPBELL, A. C., WIERNIK, G., WOOD, J., HERSEY, P., WALLER, C. A. & MACLENNAN, J. C. M. (1976) Characteristics of the Lymphopenia Induced by Radiotherapy. *Clin. Exp. Immunol.*, **23**, 200.

DI MARCO, A., DASDIA, T., GIULIANI, F., NECCO, A., CASAZZA, A. M., MORA, P. T., LUBORSKY, S. W. & WATERS, L. (1976) Biological Properties of Cell Lines Derived from Moloney Virus-induced Sarcoma. *Tumori*, **62**, 415.

EVANS, R. (1972) Macrophages in Syngeneic Animal Tumours. *Transplantation*, **14**, 468.

EVANS, R. & ALEXANDER, P. (1976) Mechanisms of Extracellular Killing of Nucleated Mammalian Cells by Macrophages. In *Immunobiology of the Macrophages*. Ed. D. S. Nelson. New York: Academic Press, p. 535.

GERAN, R. I., GREENBERG, N. H., MACDONALD, M. M., SCHUMACHER, A. M. & ABBOTT, B. J. (1972) Protocols for Screening Chemical Agents and Natural Products against Animal Tumors and Other Biological Systems. *Cancer Chemother. Rep.*, **3**, 1.

GHAFFAR, A., CALDER, E. A. & IRVINE, W. J. (1976) K Cell Cytotoxicity against Antibody-coated Chicken Erythrocytes in Tumor-bearing Mice: Its Development with Progressively Growing Tumor and the Effect of Immunization against the Tumor. *J. Immunol.*, **116**, 315.

GIULIANI, F., CASAZZA, A. M. & DI MARCO, A. (1974) Virologic and Immunologic Properties and Response to Daunomycin and Adriamycin of a Non-regressing Mouse Tumor Derived from MSV-induced Sarcoma. *Biomed. Express*, **21**, 435.

GIULIANI, F., CASAZZA, A. M., SORANZO, C. & DI MARCO, A. (1977) Effect of Pretreatment with Immune Serum on Murine Sarcoma Virus (Moloney) Tumour Induction and Growth. *Br. J. Cancer*, **35**, 190.

GIULIANI, F., SORANZO, C., CASAZZA, A. M. & DI MARCO, A. (1973) Omogenicità di Cellule Linfoidi Immuni verso il Sarcoma Murino di Moloney. *Tumori*, **59**, 269.

GREENBERG, A. H., SHEN, L. & MEDLEY, G. (1975*a*) Characteristics of the Effector Cells Mediating Cytotoxicity against Antibody-coated Target Cells. I. Phagocytic and Non-phagocytic Effector Cell Activity against Erythrocyte and Tumour Target Cells in a $^{51}$Cr Release Cytotoxicity Assay and [$^{125}$I] IUdR Growth Inhibition Assay. *Immunology*, **29**, 719.

GREENBERG, A. H., SHEN, L., WALKER, L., ARNAIZ-VILLENA, A. & ROITT, I. M. (1975*b*) Characteristics of the Effector Cells Mediating Cytotoxicity against Antibody-coated Target Cells. II. The Mouse Nonadherent K Cell. *Eur. J. Immunol.*, **5**, 474.

HERSEY, P. (1973) New Look at Antiserum Therapy of Leukaemia. *Nature*, (*New Biol.*), **244**, 22.

JOLLEY, G. M., BOYLE, M. D. P. & ORMEROD, M. G. (1976) The Destruction of Allogeneic Tumor Cells by Antibody and Adherent Cells from Peritoneal Cavities of Mice. *Cell. Immunol.*, **22**, 262.

KIRCHNER, H., HOLDEN, H. T. & HERBERMAN, R. B. (1975) Splenic Suppressor Macrophages Induced in Mice by Injection of *Corynebacterium parvum*. *J. Immunol.*, **115**, 1212.

LUNDGREN, G., ZUKOSKI, C. F. & MÖLLER, G. (1968) Differential Effects of Human Granulocytes and Lymphocytes on Human Fibroblasts *in vitro*. *Clin. Exp. Immunol.*, **3**, 817.

MACLENNAN, I. C. (1972) Antibody in the Induction and Inhibition of Lymphocyte Cytotoxicity. *Transplant Rev.*, **13**, 67.

MANTOVANI, A. (1977) *In vitro* and *in vivo* Cytotoxicity of Adriamycin and Daunomycin for Murine Macrophages. *Cancer Res.*, **37**, 815.

MANTOVANI, A., EVANS, R. & ALEXANDER, P. (1977) Non-specific Cytotoxicity of Spleen Cells in Mice Bearing Transplanted Chemically-induced Fibrosarcomas. *Br. J. Cancer*, **36**, 35.

ORTIZ DE LANDAZURI, M., KEDAR, E. & FAHEY, J. L. (1974) Antibody-dependent Cellular Cytotoxicity to a Syngeneic Gross Virus-induced Lymphoma. *J. natn. Cancer Inst.*, **52**, 147.

PERLMANN, P., PERLMANN, H. & WIGZELL, H. (1972) Lymphocyte Mediated Cytotoxicity *in vitro*. Induction and Inhibition by Humoral Antibody and Nature of Effector Cells. *Transplant. Rev.*, **13**, 91.

POLLACK, S. (1973) Specific "Arming" of Normal Lymph-node Cells by Sera from Tumor-bearing Mice. *Int. J. Cancer*, **11**, 138.

PURVES, E. C. & BERENBAUM, M. C. (1975) Selective Suppression of Murine Antibody-dependent Cell-mediated Cytotoxicity by Azathioprine. *Transplantation*, **19**, 274.

SANDERSON, C. J. & TAYLOR, G. A. (1976) Antibody-dependent Cell-mediated Cytotoxicity in the Rat. The Role of Macrophages. *Immunology*, **30**, 117.

TAGLIABUE, A., MANTOVANI, A., POLENTARUTTI, N., VECCHI, A. & SPREAFICO, F. (1977) Effect of Immunomodulators or Effector Cells Involved in Antibody-dependent Cellular Cytotoxicity. *J. natn. Cancer Inst.* (in press).

TING, A. & TERASAKI, P. I. (1974) Depressed Lymphocyte-mediated Killing of Sensitized Targets in Cancer Patients. *Cancer Res.*, **34**, 2694.

VECCHI, A., MANTOVANI, A., TAGLIABUE, A. & SPREAFICO, F. (1976) A Characterization of the Immunosuppressive Activity of Adriamycin and Daunomycin on Humoral Antibody Production and Tumor Allograft Rejection. *Cancer Res.*, **36**, 1222.

VOSE, B. M. & MOUDGIL, G. C. (1976) Post-operative Depression of Antibody-dependent Lymphocyte Cytotoxicity following Minor Surgery and Anaesthesia. *Immunology*, **30**, 123.

ZIGHELBOIM, J., BONAVIDA, B. & FAHEY, J. L. (1973) Evidence for Several Cell Populations Active in Antibody Dependent Cellular Cytotoxicity. *J. Immunol.*, **111**, 1737.

ZIGHELBOIM, J., BONAVIDA, B. & FAHEY, J. L. (1974) Antibody-mediated *in vivo* Suppression of EL4 Leukemia in a Syngeneic Host. *J. natn. Cancer Inst.*, **52**, 879.